# Audiovisual structural connectivity in musicians and non-musicians: a cortical thickness and diffusion tensor imaging study

**DOI:** 10.1038/s41598-021-83135-x

**Published:** 2021-02-22

**Authors:** Cecilie Møller, Eduardo A. Garza-Villarreal, Niels Chr. Hansen, Andreas Højlund, Klaus B. Bærentsen, M. Mallar Chakravarty, Peter Vuust

**Affiliations:** 1 Center for Music in the Brain, Department of Clinical Medicine, Aarhus University & The Royal Academy of Music, Aarhus/Aalborg, Universitetsbyen 3, Building 1710, 8000 Aarhus C, Denmark; 2 Center of Functionally Integrative Neuroscience, Aarhus University, Aarhus, Denmark; 3 Institute of Neurobiology, Universidad Nacional Autónoma de México, Boulevard Juriquilla 3001, C.P. 76230 Querétaro, Querétaro Mexico; 4 Aarhus Institute of Advanced Studies, Aarhus University, Aarhus, Denmark; 5 Interacting Minds Centre, Aarhus University, Aarhus, Denmark; 6 Department of Psychology, Aarhus University, Aarhus, Denmark; 7 Cerebral Imaging Center, Douglas Mental Health University Institute, Montreal, QC Canada; 8 Department of Psychiatry, McGill University, Montreal, QC Canada; 9 Department of Biological and Biomedical Engineering, McGill University, Montreal, QC Canada

**Keywords:** Neuroscience, Auditory system, Cognitive neuroscience, Sensory processing, Visual system

## Abstract

Our sensory systems provide complementary information about the multimodal objects and events that are the target of perception in everyday life. Professional musicians’ specialization in the auditory domain is reflected in the morphology of their brains, which has distinctive characteristics, particularly in areas related to auditory and audio-motor activity. Here, we combined *diffusion tensor imaging* (DTI) with a behavioral measure of visually induced gain in pitch discrimination, and we used measures of *cortical thickness* (CT) correlations to assess how auditory specialization and musical expertise are reflected in the structural architecture of white and grey matter relevant to audiovisual processing. Across all participants (*n* = 45), we found a correlation (*p* < 0.001) between reliance on visual cues in pitch discrimination and the *fractional anisotropy* (FA) in the left *inferior fronto-occipital fasciculus* (IFOF), a structure connecting visual and auditory brain areas. Group analyses also revealed greater cortical thickness correlation between visual and auditory areas in non-musicians (*n* = 28) compared to musicians (*n* = 17), possibly reflecting musicians’ auditory specialization (FDR < 10%). Our results corroborate and expand current knowledge of functional specialization with a specific focus on audition, and highlight the fact that perception is essentially multimodal while uni-sensory processing is a specialized task.

## Introduction

Perception in everyday life is essentially multimodal. Recent years have witnessed an increasing interest in multisensory processing^1^ which has resulted in accumulating evidence of crosstalk between primary sensory areas of the brain, previously thought to be modality-specific^2–4^ and their perceptual and behavioral correlates^5,6^. Not only do such findings refine the existing knowledge of multisensory convergence sites in higher-level cortical areas such as the superior temporal sulcus^7,8^, the prefrontal cortex^9^, and the association cortices^10^. The findings also foster enhanced interest in exploring putative mechanisms underlying multisensory processing, and they challenge existing theoretical accounts of perceptual processing and encourage the development of new ones.

Within the last couple of decades, musicians have become a desired model of structural brain plasticity^11^. Possibly due to lifelong musical training, the morphology of musicians’ brains differ systematically from non-musicians’^12–14^, particularly in auditory^15–17^ and motor^18,19^ areas. Studies using *diffusion tensor imaging* (DTI) have revealed variations in the *fractional anisotropy* (FA) of the corticospinal tract associated with musicianship^20^ and variations in the FA of the superior longitudinal fasciculus associated with different levels of musical expertise^21^. Bermudez and colleagues^22^ found that musicianship correlated with greater cortical thickness and volume in temporal and frontal regions. They also found that musicians showed more localized structural whole-brain covariance than non-musicians. It is commonly agreed that musical activities require sophisticated dynamic interplays between multisensory and motor behaviors, sub-served by particularly the auditory, visual, tactile, and motor systems of the brain^12,14,23^. Notwithstanding, only little research has been conducted that specifically assesses the plasticity of audiovisual regions of the brain and how it relates to musical training. A recent review on musicians as a model of brain plasticity highlights the strengthening of connections between multisensory brain areas that is associated with musical training, but does not reference studies that directly investigate such connections between auditory and visual brain areas^11^. As the first of its kind, a magnetoencephalography (MEG) study by Paraskevopoulos and colleagues^24^ investigated the cortical functional network associated with audiovisual integration and its reorganization accompanying musical training. They concluded that non-musicians rely on processing of visual information, whereas musicians showed enhanced functional connectivity primarily in auditory regions. Exploratory analyses of our recent behavioral study support a similar conclusion^25^, by suggesting that non-musicians benefit more from visual cues in a pitch discrimination task.

To our knowledge, no study has yet investigated musician-specific characteristics of audiovisual connections in the brain. Hence, the purpose of the present study was to assess how auditory specialization and musical expertise are reflected in the structural architecture of white and grey matter involved in audiovisual processing. To this aim, we used DTI in combination with a behavioral measure of auditory specialization, operationalized as visually induced gain in pitch discrimination^25^. Specifically, in an oddball paradigm, we measured the extent to which cross-modally matching visual cues aided detection of subtle pitch changes (± 20 and 30 cents). Visual stimuli consisted of a disc placed below, in, or above the center of the screen. Responses in the crossmodally matching condition were compared to the condition in which the visual information did not provide information about the pitch change (see “Materials and methods” and Supplementary Information, Fig. S1). This difference measure, termed bimodal compatibility gain (BCG), was used in the analysis of the DTI data across all participants (*n* = 45) to assess how relative reliance on visual information in an auditory task is reflected in white matter connections between auditory and visual brain areas.

Grey matter characteristics of musical expertise were assessed with group analyses of cortical thickness (CT), using the analysis technique *Mapping anatomical correlations across cerebral cortex* (MACACC) to measure structural covariance^26^. The MACACC analysis is performed by selecting a seed vertex of interest from the cortical surface map, and correlating the cortical thickness (CT) of this seed with the CT of all other brain vertices. This approach is similar to seed-based functional connectivity analysis, which correlates a measure (i.e. the BOLD signal) in a region of interest (ROI) with the whole brain^27–29^, and has been used to show differences in cortical thickness covariance between musicians and non-musicians^22^. The resulting statistic gives an indication of the degree to which CT throughout the brain covaries with the seed region across participants. It is not a direct measure of connectivity by any means, but it can insinuate about the possible structural relationship between different areas.

Focusing our region of interest analysis on the bilateral inferior fronto-occipital fasciculus (IFOF), we hypothesized that participants (*n* = 45) who benefit more from visual cues in the behavioral pitch discrimination task regardless of their musical background would show larger FA values in this structure, which is known to connect auditory and visual areas of the cortex^30^. Furthermore, based on Bermudez and colleagues'^22^ structural covariance results, we expected to find greater cortical thickness correlation between visual and auditory areas (V1 and Heschl’s gyrus) in non-musicians (*n* = 28) than professional musicians (n = 17), suggesting stronger audiovisual connectivity. This MRI/DTI study is part of a larger study which includes the published paper mentioned above^25^. See Supplementary Information, Supplementary Introduction for further details.

## Results

### Behavioral results

To quantify the beneficial effect of relevant visual information on subtle pitch change detection, referred to as the bimodal compatibility gain (BCG), we first calculated the sensitivity index, *d′*, for each participant in each experimental condition using the formula: *d′* = *Z*(hit rate) − *Z*(false alarm rate)^31^. BCG was quantified by subtracting the mean *d’* across the two pitch levels in the “no visual cue” condition from that of the “crossmodally matching” condition of the behavioral experiment, separately for each participant.

**Figure 1:**
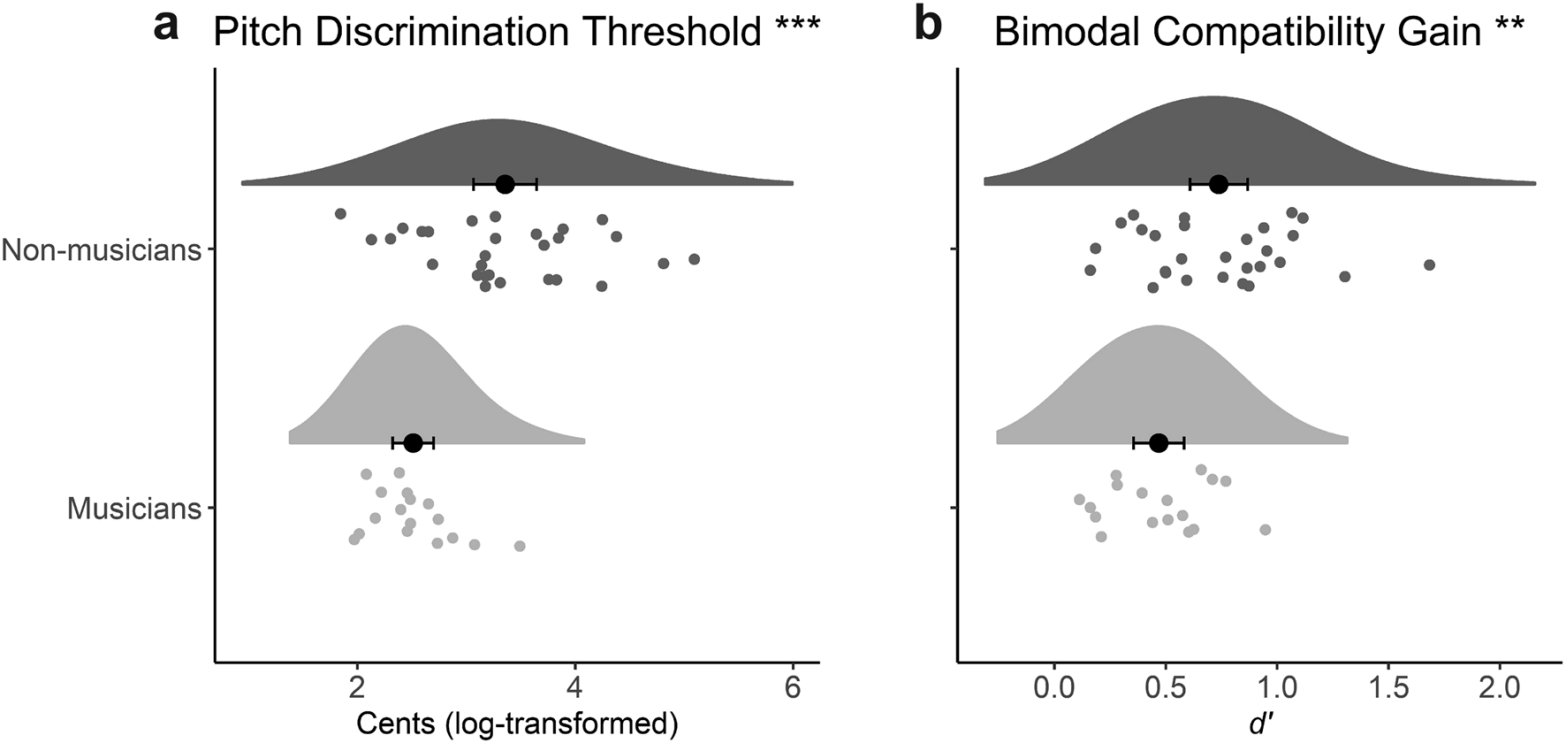
Means of the (**a**) log-transformed Pitch Discrimination Thresholds (PDT) and the (**b**) Bimodal Compatibility Gain (BCG), (i.e. crossmodally matching condition minus no visual cue condition) are significantly higher in non-musicians than in musicians. Error bars represent 95% confidence intervals. Asterisks indicate significant differences between groups, ***p < 0.001, **p < 0.01.

We ran assumption tests on the two behavioral variables, i.e., pitch discrimination thresholds (PDT) and the BCG. Outliers and non-normally distributed PDT scores were resolved with a log-transformation. Shapiro–Wilk tests confirmed normal distributions of the log-transformed PDT as well as BCG in professional musicians (MUS) (PDT: *W* = 0.942, *p* = 0.347; BCG: *W* = 0.964, *p* = 0.709) and non-musicians (NM) (PDT: *W* = 0.980, *p* = 0.845; BCG: *W* = 0.967, *p* = 0.482). Bartlett’s tests confirmed homogeneity of variances in the two groups in the case of the BCG (*B* = 2.610, *p* = 0.106), but not in the case of the PDT (*B* = 7.687, *p* = 0.006). As is clearly visible in the Raincloud plots^32^ of Fig. 1, there was a greater distribution of scores in the NM group. Therefore, differences between MUS and NM were assessed with Welch t-tests, which do not assume equal variances. Behavioral results confirmed that the PDT of NM were significantly higher (poorer) than those of MUS, *t*(42) = 4.79, *p* < 0.001. Importantly, NM responses in the behavioral experiment were also significantly more influenced by the visual stimuli than the responses of the MUS group, as evidenced by larger BCG scores, *t*(42.3) = 3.06, *p* = 0.004, (see Fig. 1 for descriptive statistics).

### DTI

**Figure 2:**
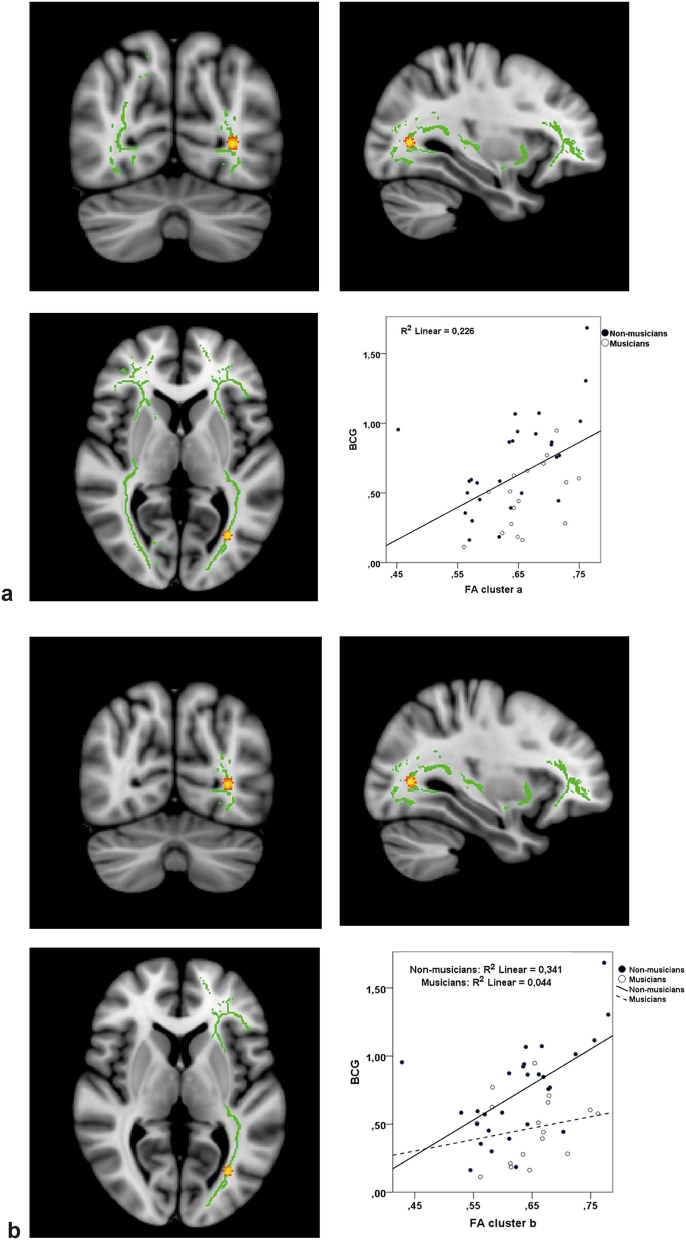
Significant clusters and corresponding scatter plots. (**a**) Across groups. Significant cluster resulting from correlating bimodal compatibility gain (BCG) with the FA value of the ROI mask voxels, using age, gender and group as covariates. The scatterplot shows a positive correlation in this area. Threshold-free cluster enhancement was used to control for multiple comparisons with family-wise error (FWE) at alpha < 0.05. (**b**) Separate analyses of musicians and non-musicians with (FWE) at alpha < 0.10. In non-musicians only, a significant cluster was found, which overlapped and was extended in size compared with the original cluster. Musicians are included in the scatterplot, but the brain images depict only non-musicians.

The *tract-based spatial statistics* (TBSS) analysis of the DTI data showed no significant differences between musicians and non-musicians. However, across all participants (*n* = 45) we found a significant FA cluster related to the BCG in the occipital part of the left inferior fronto-occipital fasciculus (IFOF) (peak, x = − 31, y =  − 68, z = 5, size = 3, *t* = 3.38, *p* < 0.001). There was a positive association between BCG and FA in that region (Fig. 2a). We also ran the TBSS analysis on the two groups separately and only in the left IFOF, i.e. the hemisphere of the original cluster. In this analysis, the alpha level was adjusted to < 0.1 with FWE correction for multiple comparisons. We found a significant FA cluster (peak, x = − 31, y = − 70, z = 5, size = 6, *t* = 3.94, *p* = 0.09) in the NM group only. This NM-cluster overlapped with the original one, and here the BCG was also positively associated with the FA value (Fig. 2b). In comparison, the association between BCG and FA in the left IFOF of the MUS group was not statistically significant (*p* = 0.64).

### Anatomical correlations analysis (MACACC)

**Figure 3:**
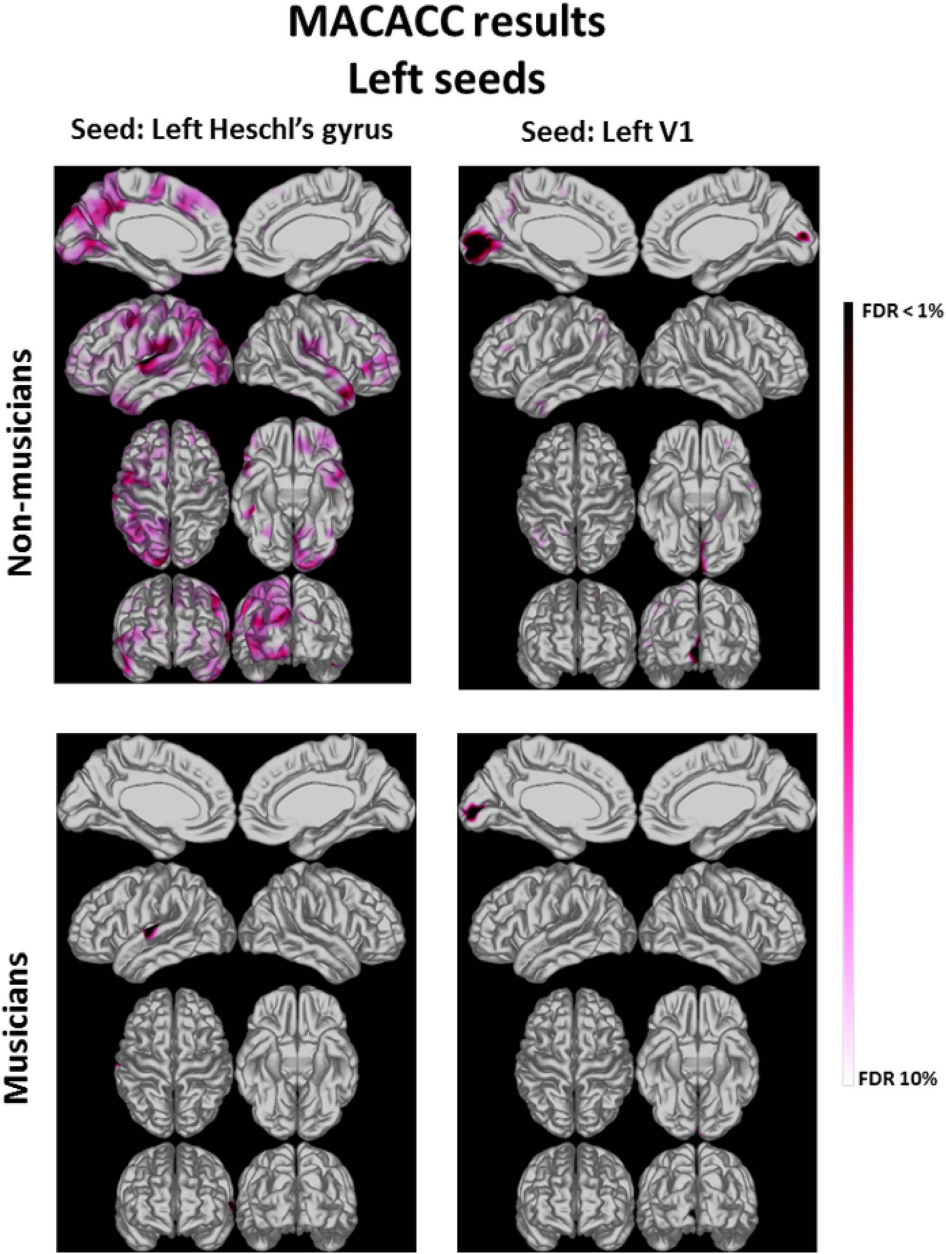
Significant correlations between the cortical thickness (CT) of vertices of the whole brain and vertices of seeds in left Heschl’s Gyrus (left column) and left V1 (right column). FDR = False discovery rate.

**Figure 4:**
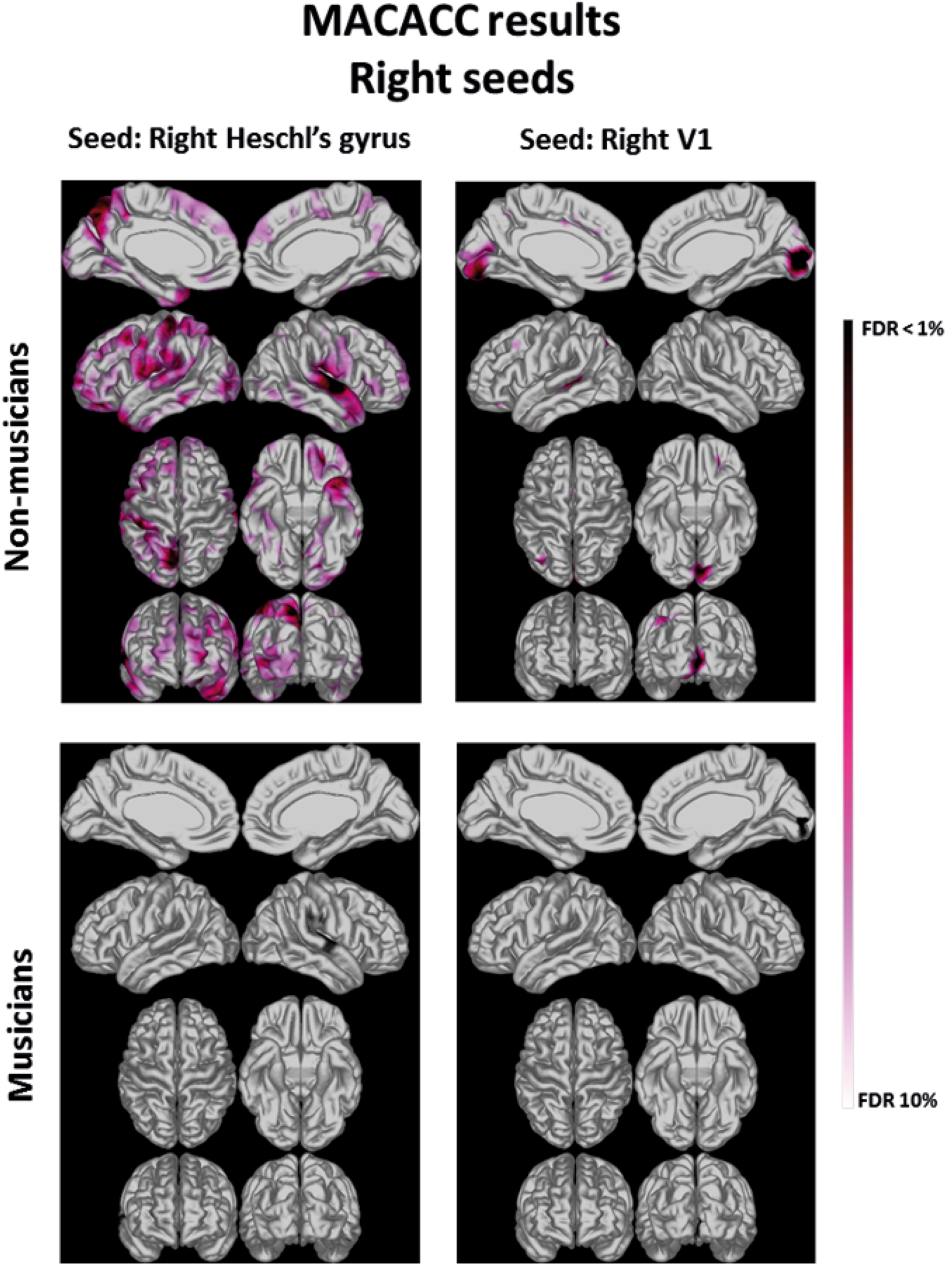
Significant correlations between the cortical thickness (CT) of vertices of the whole brain and vertices of seeds in right Heschl’s Gyrus (left column) and right V1 (right column). FDR = False discovery rate.

As expected, all four seeds (bilateral Heschl’s gyrus and V1) showed significant CT correlation with the locally surrounding gray matter. Interestingly, the Heschl’s gyrus seeds showed significant correlations distributed across the whole brain in NM but not in MUS (see significant correlations in Figs. 3 and 4. See all correlations in Fig. S2 and Fig. S3 of the Supplementary Information). The V1 seeds also showed significant correlations distributed across the whole brain in NM but not in MUS. Importantly, in the NM group, Heschl’s gyrus seeds were correlated with visual areas within the same and opposite hemispheres, and V1 seeds were correlated with auditory areas, mostly within the left hemisphere (Supplementary Information, Table S1). These diverging results were found despite no significant group differences in CT of any of the seeds (*p* > 0.05 for all four seeds), as assessed with a Mann–Whitney test. To exclude the possibility that power differences account for the diverging MACACC analysis findings for the MUS and NM groups, we performed a control analysis on a random subset of the NM group that was comparable in size to the MUS group (*n* = 17) and found similar results. The MACACC-slope group analysis did not show significant differences between groups.

## Discussion

The auditory specialization associated with musical expertise has ramifications for brain and behavior. In this study we related behaviorally measured reliance on visual cues for pitch discrimination, i.e., the bimodal compatibility gain (BCG), with *fractional anisotropy* (FA) values within the inferior fronto-occipital fasciculus (IFOF), a white matter tract that connects auditory and visual brain areas^30^. With seeds in auditory and visual areas, we also analyzed cortical thickness correlations with all other vertices of the brain across groups of non-musicians and expert musicians.

The behavioral data indicated that when asked to perform the same subtle pitch discrimination task, the performance of musicians who are highly specialized within the auditory domain improves less with the help from visual cues than the performance of non-musicians, as suggested by the significantly smaller BCG in musicians. Across groups, higher BCG, in turn, was related to higher FA values in the left IFOF. Interestingly, separate analyses of the two groups revealed a significant cluster in non-musicians only. This cluster overlapped with the original cluster, yet was extended in size. To our knowledge, this is the first study to link structural neuroanatomy to behavioral audiovisual performance in musicians and non-musicians. Note, though, that the statistical threshold of the additional separate groups analyses was increased from 0.05 to 0.1, to account for the smaller sample sizes.

These DTI results were corroborated by our analyses of cortical thickness correlations that we performed using the technique MACACC^26^. With seeds in Heschl’s Gyrus and in V1 we show that the topology of the cortical thickness correlations is different in the two groups. The non-musicians show a more distributed pattern that includes visual and auditory areas with both seeds, whereas musicians show only local correlations, suggesting cortical specialization.

Inter-individual variability in multisensory behavior can readily be linked to microstructural characteristics of white matter pathways connecting the relevant sensory systems, as was recently demonstrated within the visuo-haptic domain^33^. By focusing our analysis specifically on the inferior fronto-occipital fasciculus (IFOF) we tested the hypothesis that participants who benefit more from visual cues in a behavioral pitch discrimination task show larger FA values in the white matter pathway that traverses both visual and auditory regions of the brain. This approach is similar to the one used by Zamm and colleagues^34^ who studied colored-music synesthesia, a form of synesthesia where musical sounds elicit colored percepts. They found evidence of stronger connections between visual and auditory association regions in people with colored-music synesthesia than in matched controls. In the case of synesthesia, a clear-cut categorical definition of the phenomenon and its neurobiological foundation is difficult to attain because sensory-specific features of different modalities are associated, albeit to a less radical extent, in non-synesthetes as well^35,36^. Indeed, the audiovisual stimuli used in the present behavioral experiment were selected by virtue of their crossmodal correspondence, which is well-established in the general population^37–40^. Hence, though the task and population studied by Zamm and colleagues^34^ were different to those of the present study, the similarities are worth exploring. With voxelwise comparisons within the IFOF, Zamm and colleagues found a linear association between behavioral Synesthesia Battery scores^41^ and FA values in a significant cluster consisting of seven voxels, which was located in white matter in fusiform gyrus within the posterior portion of the right IFOF. In comparison, our regression analyses of FA values on audiovisual behavioral scores in all participants also revealed a single significant cluster in a location of the IFOF remarkably similar to the one found by Zamm and colleagues, however in the opposite hemisphere. They argued that their finding may support the notion that synesthetes and non-synesthetes lie along the same neurologically defined continuum, though more research is needed to answer this yet unresolved question conclusively^42,43^. Indeed, our findings support the existence of a continuum, which describes the extent to which visual features influence auditory perception, and which is sub-served by the posterior portion of the IFOF. Importantly, though, our TBSS analyses performed on the two groups separately revealed a significant cluster related to the behavioral measure only in non-musicians. In light of this finding, it would be relevant for future studies on structural correlates to synesthesia to investigate the possibility of dissociating synesthetes with musical training from those without. This was not an option in the study by Zamm and colleagues as they only assessed a limited range of musical experience, and their groups were carefully matched on musical training. Likewise, including measures of synesthetic behavior, e.g. obtained via the Synesthesia Battery^41^, in studies on audiovisual perception in musicians and non-musicians could cast light on the possible interaction of musicianship on the putative neurologically defined continuum.

When interpreting the results of our group analyses involving the BCG, one could be concerned that the size of the BCG would be limited by (superior) pitch discrimination abilities, in which case musicians would show smaller BCGs by virtue of their smaller PDTs per se and not by how much they make use of visual cues in pitch discrimination. To assess whether this is the case here, we have included a scatter plot in Supplementary Information (Figure S6) showing PDT plotted against BCG separately for all musicians (n = 17) and for those non-musicians (n = 17) who have PDTs in a similar range as the musicians. The logic here is that if musicians are up against hard biological limits in human pitch discrimination, musicians with the lowest PDTs will also show the smallest BCGs. The plot suggests, however, that while the PDT is linked to BCG in non-musicians, such a relationship is not apparent in musicians and hence the BCG does not appear to be limited by their (low) pitch discrimination thresholds.

Our behavioral findings that participants with larger (poorer) pitch discrimination thresholds benefitted more from the visual cues in the experiment as evident by larger BCG is consistent with the prediction of the *principle of inverse effectiveness* (PoIE). This basic principle, which governs multisensory processing at the single-neuron level, expresses how spatio-temporally coinciding stimuli from different modalities have maximal synergistic effects when responses to the unimodal constituents of the stimuli are weak^44,45^. It was initially derived from studying response properties of single neurons in the cat superior colliculus^45^ and, similarly, most subsequent work has utilized animal models^46–48^. Investigations designed to assess whether the principle governs the functional level of neuronal responses in humans are few^8^, despite growing interest in the extent of its application to human behavior^25,49–53^. Within the auditory and visual domains, studies have shown effects of the PoIE at early stages of cortical processing in humans^4,54,55^. We found that behaviorally measured pitch discrimination thresholds are inversely related to visually induced gains, which in turn is related to the FA value in a significant cluster in the left IFOF. To our knowledge, the present study is the first to show structural correlates of a behavioral measure of visually induced gains in pitch discrimination whose between-subject variability can be accounted for in terms of inverse effectiveness.

A note on the meaningfulness of the observed left-lateralization is in place, as the task was pitch-related and one might expect it to be relatively more right auditory cortex dependent owing to the known right hemisphere dominance in pitch perception^56^. However, the measure derived from our task, i.e., the bimodal compatibility gain, is not in itself a measure of pitch discrimination but rather a measure of the extent to which visual information influences auditory perception. While we used a pitch-related task in the present study and found left IFOF involvement, it is likely that measures of bimodal compatibility gain derived from auditory tasks involving other features, e.g., intensity, timbre, rhythm, location etc., would exhibit a high within-subject correlation with each other and as such would show similar left IFOF involvement. In other words, the bimodal compatibility gain is not necessarily tied to the right hemisphere as is pitch perception per se, but could be related to more general auditory sensitivity, particularly in light of the PoIE described above. This is of course still speculation and should be tested in future studies.

To complement the ROI analysis of our DTI data, we performed whole-brain cortical thickness correlation analyses, using the technique MACACC^26^. Our hypothesis rested on the assumption that functional specialization leads to anatomical change in areas of the brain that subserve the function in question^26^. Consistent with our hypothesis, the analysis revealed that non-musicians show greater cortical thickness correlations between visual and auditory areas than musicians, who show significant correlations primarily in the locally surrounding grey matter of the selected seeds. While these structural results should not be interpreted as functional evidence that these areas are used in the present audiovisual task, it is indeed consistent with our finding that non-musicians gain more from the visual cues in the pitch discrimination task, and that the size of the gain is reflected in the FA value of the significant cluster within the IFOF.

Our findings regarding differences in the structural audiovisual connectivity of musicians and non-musicians are also compatible with functional evidence that musicians recruit a sparser network of the brain mainly centered on auditory cortices in an audiovisual task, while the cortical network recruited by non-musicians include visual and other non-auditory regions of the brain^24^. More generally, our findings extend previous evidence derived from MACACC analyses of greater specificity in musicians in the relationship between thickness of frontal and auditory regions^22^, areas known to be coactivated in music-related tasks^57^. In the analysis performed by Bermudez and colleagues^22^, seeds were in those areas of the right frontal cortex that exhibited greater cortical thickness in musicians than non-musicians. Specifically interesting in the context of the present study, Bermudez and colleagues also found that the significant correlations in non-musicians were much more expansive than in musicians. This pattern corresponds well with our results, although their analysis also revealed significant correlations outside the locally surrounding grey matter in musicians. As discussed below, this discrepancy may be explained by the greater statistical power of their analysis compared to what we could obtain with our limited sample sizes.

It could be argued that the lack of correlations between CT in Heschl’s gyrus and elsewhere in musicians found in the present study could simply be an artefact arising from the possibility that musicians’ auditory cortices are larger than non-musicians’. This would automatically decrease the correlation with CT in musically unrelated areas, which would be a secondary effect that has little to do with differences in audiovisual processing between musicians and non-musicians. However, while other studies have indeed shown significantly larger auditory cortices in musicians^16,56^, we found no significant differences between our groups for any of the seeds used in the MACACC analysis. Hence, for the present data, the diverging results in the two groups cannot be attributed to differences in CT of the seeds used.

We found that the visually-induced gain in pitch discrimination was considerably larger in our non-musician sample. This result may well be viewed in light of recent theoretical and empirical advances within the field of perceptual narrowing that explain effects of early experience on multisensory perceptual expertise not only in terms of conventional perceptual *broadening* but also highlighting *narrowing* as a prominent mechanism underlying perceptual development^58^. The concept of unisensory narrowing emerged from studies that showed how infants’ responsiveness to perceptual inputs is initially broad, encompassing native as well as non-native sounds and sights. From the second year of life, however, there is a radical decrease in infants’ ability to detect violations of patterns not associated with their particular ecological setting. As such, perceptual narrowing marks the beginning of perceptual specialization and expertise.

Particularly relevant in the context of the present study, it appears that perceptual narrowing generalizes to multisensory perceptual development. In the beginning of life, infants attend primarily to the eyes of a person speaking to them^59,60^, yet it was recently shown that by six months of age, they attend equally to the eyes and to the mouth, i.e. the source of the speech signal. Two months later, i.e. as the characteristic baby babbling emerges, they attend primarily to the mouth and continue doing so until the age of 12 months when they shift back to focusing primarily on the eyes of someone speaking their native language, a strategy also observed in adults^61^. This shift is seen as an indication that infants are now becoming so familiar with their native language that they no longer need the redundant information provided by the lips of the talker, but can attend to the social cues provided by the eyes. Interestingly, children aged 12 months that are exposed to non-native audiovisual speech will continue to pay closer attention to the mouth, presumably because the bimodal information aids their quest to make sense of the unfamiliar speech signal^58^.

A similar account may explain non-musicians’ increased reliance on visual cues in the present study. Musicians’ specialized auditory ecology makes them on home ground in a pitch discrimination task. In contrast, explicit pitch discrimination is not part of daily activities for non-musicians and hence such a strictly auditory task may appear unfamiliar. Extending this idea to the language domain, we may propose that to a performing musician, music is like a native language in which pitch discrimination and production are meaningful, comparable to phoneme discrimination and production in a native language. Indeed, comparative work on music and language has likened music making to communication^62^, and it has been shown that jazz musicians’ pre-attentive brain responses to rhythmic incongruence are left-lateralized, indicating functional adaptation to a communicative task similar to language^63^. Hence, in perceptual narrowing terms, although the visual cues available in the experiment carry information about the changes in pitch that are relied upon by non-musicians unfamiliar with the musical language, the expert musician considers them redundant.

The present study has some limitations. The results of the TBSS analyses on the two groups separately must be interpreted in light of the fact that we increased the statistical threshold to alpha = 0.1, with family-wise error (FWE) correction for multiple comparisons. This was preferred because the original cluster only consisted of three voxels and we expected a similarly small effect, which would be harder to detect when splitting the sample in two due to the corresponding decrease in statistical power. Hence, had we maintained the statistical threshold at alpha = 0.05, we would increase the risk of not finding an effect present in the data (and hence commit a type II error). In our view, this approach is further warranted by the substantial difference found between the two groups, i.e. the fact that the cluster was increased in size and significant at *p* = 0.09 in non-musicians whereas the mean FA-value of the same voxels in musicians was far from significantly related to the behavioral score (*p* = 0.64).

Likewise, in the MACACC analysis, our significance threshold for the multiple comparisons FDR was set at 10% (*q* = 0.1), thus allowing more exploration of the whole cortex. We have successfully used this procedure in our previous studies^64^. This more liberal statistical analysis strategy warrants caution when interpreting the findings. The lack of significance in the MACACC-slope analysis between groups is an important cautionary result that could be related to the small sample size.

Visual inspection of the plots in Fig. 2 suggests that an outlier was present in the data. One non-musician participant’s FA value in the significant cluster found in analysis a) was 2.956 *SD*s from the mean FA of all participants (see outlier diagnostics in Supplementary Information, Fig. S4). Removing this outlier, however, did not have detrimental effects on the reported results. In fact, doing so may rather result in a steeper slope of the fit line representing the relationship between BCG and FA in non-musicians (see Supplementary Information, Fig. S5).

TBSS is a rough indirect measure of white matter integrity with many drawbacks^65^ that should also be considered when interpreting the present results. Further research using better measures of white matter integrity that make use of multi-shell acquisitions (i.e. constrained spherical deconvolution) should help confirm these findings.

Finally, our recruitment included professional musicians because musicians show superior sensitivity to auditory-only pitch changes at behavioral as well as neural levels^66,67^. We did not collect information on a number of factors that may additionally influence basic pitch discrimination, incl. general intelligence, age of onset of formal musical training, tonal language skills, and absolute pitch, the rare ability enabling people to assign names to specific tones even in the absence of a reference tones. The focus here was on expertise-related variations in the use of visual perceptual information in subtle pitch discrimination. We did not seek to disentangle our notion of musical expertise from the above factors although this could be an interesting scope in future studies of visual influences on pitch discrimination, where controlling for such co-varying factors could potentially improve the precision of the conclusions.

In sum, recognizing the fact that multisensory processing is fundamental to perception, the study reported here investigated neuroanatomical correlates to differences in the extent to which musicians and non-musicians make use of visual cues in pitch discrimination. Our findings makes an important contribution to the existing literature showing that the specialized auditory skills associated with intense musical training are reflected in grey and white matter anatomy of the human brain, specifically by suggesting weaker interdependence of auditory and visual areas of the brain in musicians than in non-musicians. In the broader perspective, we believe that these findings have implications for the generalizability of perceptual skills assessments for research as well as for clinical purposes when performed on sensory systems in isolation from each other. Our study shows that crossmodal information is relevant and valuable even to so-called unimodal perception, and particularly so for the unspecialized whose brains have adapted to an ecological setting in which rich multisensory information is not only continuously present but also relied upon.

## Materials and methods

### Participants

The recruitment strategy supported inclusion of participants with a wide range of pitch discrimination abilities who were able to perform the difficult pitch discrimination task. Seventeen musicians (MUS) (9 males, mean age 24.1, *SD* 4.1) and 30 non-musicians (NM) (13 males, mean age 24, *SD* 3.3) with normal or corrected-to-normal visual acuity and no hearing impairments volunteered to participate in the study, which was conducted in Danish. Participants with no hits to any of the largest pitch deviants (30 cents) in any one of the five blocks (see below) were subsequently excluded. This was the case for two NM (one male, one female). More non-musicians than musicians were initially recruited because we expected that more non-musicians were unable to perform the task.

**Figure 5:**
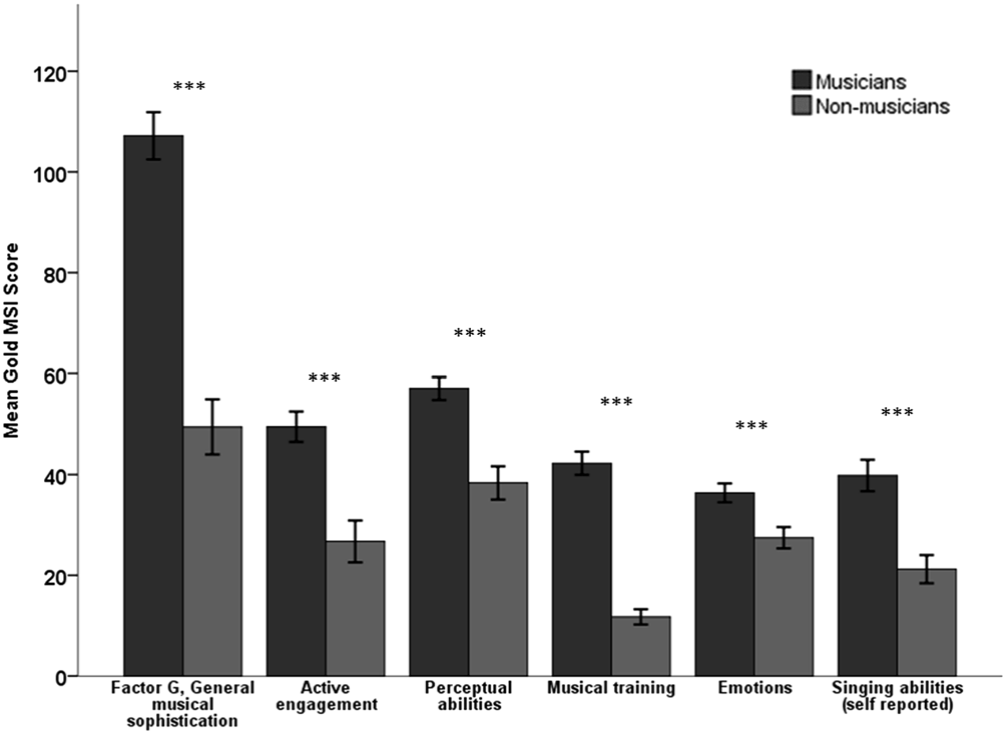
Scores on the six subscales of Goldsmiths Musical Sophistication Index, (Gold MSI), v.1.0. Independent t-tests showed statistically significant differences between the groups for all subscales, ***p < 0.001. Error bars indicate 95% confidence intervals.

The MUS group consisted of full-time conservatory students or professional musicians, and the NM group had no formal music training and no experience with playing musical instruments including singing beyond mandatory primary school music lessons. A Danish translation (available online at http://www.gold.ac.uk/music-mind-brain/gold-msi/download) of the questionnaire part from *Goldsmiths Musical Sophistication Index*, v.1.0 (Gold MSI)^68^ assessed participants’ self-reported level of musical experience and sophistication. Figure 5 shows how MUS scores were significantly higher than NM scores on all subscales of the instrument.

Before the experiment, participants gave their written informed consent. They received financial compensation (DKK 400,-) for participating in the study, which took place on two separate days in Aarhus, Denmark. The study protocol was approved by The Central Denmark Regional Committee on Health Research Ethics (Project-ID: M-2014-52-14) and the study was conducted in accordance with the guidelines from the Declaration of Helsinki.

### Behavioral experiment

The behavioral experiment was conducted 2–4 weeks before the MRI session, and it is reported in full in Møller et al.^25^. Briefly, participants were equipped with headphones (Sennheiser HDA200) and seated in front of a computer screen (refresh rate: 60 Hz) in a sound-attenuated, normally lit behavioral lab. Sinusoidal tones of 523.25 Hz (corresponding to C5 in Scientific Pitch Notation) served as standard tones in an oddball paradigm. Target deviant tones consisted of two levels of pitch change (20 cents and 30 cents) deviating in two directions (high and low relative to the standard). The purpose of including two levels was to increase the sensitivity of the paradigm to responders with both high and low pitch discrimination thresholds. Participants’ task was to fixate on the center of the screen, and to report detection of deviant tones by pressing the space button of the keyboard as fast as possible without making mistakes. The screen displayed visual images of a light grey disk on a dark grey background. The disc was positioned above, below, or in the center of the screen (see Supplemental Information, Fig. S1). A standard audiovisual stimulus comprised a standard tone coupled with a disc in the center position. Because responses did not differ between high and low deviants, these two conditions were concatenated. When following the rule “higher pitch corresponds to higher vertical position”^39^, this resulted in two levels (20 cents and 30 cents) of three categories of audiovisual target deviant stimuli: “crossmodally matching” (e.g. high pitch/high position), “crossmodally mismatching” (e.g. high pitch/low position), and “no visual cue” (i.e. high or low pitch/center position). The oddball paradigm contained 80% standards, and the deviants were pseudo-randomly presented with 3–7 standards in between, using Presentation software (Neurobehavioral systems Inc., Albany, CA, USA). The duration of the auditory stimuli was 100 ms, including 5 ms fade in/out, with an inter-stimulus interval (ISI) of 700 ms. The duration of the visual stimuli, which were presented simultaneously, was 800 ms, i.e. with no ISI. Auditory stimuli were delivered at approximately 70 dB SPL. A ~ 1-min training block, otherwise identical to the experimental blocks, preceded the experiment. Four experimental blocks with 40 trials per condition (4 min 40 s duration) and one auditory only control block (5 min 20 s duration) randomly positioned as no. 2, 3, or 4 of the five blocks were featured. Participants were given 1-min breaks in between blocks, and the total duration of the behavioral experiment was ~ 30 min.

Responses with latencies between 200 and 1000 ms after stimulus onset were included in the analysis of the experiment. Data from the behavioral experiment were preprocessed in IBM, SPSS Statistics, 24. R^69^, version 4.0.2, was used for the statistical analyses and plotting of the pitch discrimination thresholds and bimodal compatibility gain.

### Pitch discrimination threshold (PDT) estimation

Using a *two-down, one-up* adaptive staircase procedure^70^, individual pitch discrimination thresholds (PDT) were measured 2–4 weeks after the experiment on the day of the MRI acquisition. The staircase procedure employed a criterion-free AXB forced-choice task and was adapted from Williamson et al.^71^ to match the stimuli and participants of the present study. The duration of the tones was 100 ms and the ISI was 300 ms. The reference frequency was always 523.25 Hz and participants’ task was to state whether the first (A) or the last (B) tone differed from the two other tones. The staircase terminated after 14 reversals and the pitch discrimination threshold (PDT) was calculated on the basis of an average of the last six reversals. The duration was approximately three to five minutes depending on participants’ individual response times.

### MRI acquisition

The MRI data were collected on the same day as the PDT, using a Siemens Magnetom Skyra 3 T scanner and a 32-channel head coil. We acquired the following sequences: (1) T1-weighted single-shot MPRAGE 3D sagittal with FOV = 256, matrix = 256 × 256, gap = 0, slices = 176, TR/TE = 2300/3.8 ms, FA = 8o, direction = ascending, voxel = 1 mm^3^ filters = Distortion Correction (2D), prescan normalize, elliptical filter. (2) DTI (blip down) spin-echo with FOV = 210 mm, matrix = 140 × 140, slices = 70, orientation = transversal, direction = interleaved, phase-encoding direction = A > P, voxel = 2 mm^3^, TR/TE = 9200/86 ms, fat suppression = strong, GRAPPA acceleration × 3, b0 = 9 volumes, b-value   =  1500 s/mm^2^, directions = 62.  (3)  DTIPA (blip up), spin-echo with FOV = 210 mm, matrix = 140 × 140, slices = 70, orientation = transversal, direction = interleaved, phase-encoding direction = P > A, voxel = 2 mm^3^, TR/TE = 9000/84 ms, fat suppression = strong, GRAPPA acceleration × 3, b0 = 4 volumes. The DWI sequence was used as the opposite blip for field mapping using FSL-topup (see “DTI preprocessing, processing, and statistics”).

### T1-weighted image preprocessing and processing

The T1-weighted images were converted from DICOM to MINC format for preprocessing. The images were preprocessed using an in-house Bpipe-based preprocessing pipeline (http://cobralab.ca/software/mincbeast_bpipe.html) that uses the MINC Tool-Kit and the ANTs software^72^. We performed the following preprocessing steps: N4 bias field correction^73^, linear registration to MNI-space, cut-neck (we cropped the region around the neck), and transformation back to native space. The preprocessed images were then fed to the cortical thickness pipeline. Cortical thickness (CT) was estimated using the CIVET processing pipeline (version 1.1.12; Montreal Neurological Institute). Specifically, all T1-weighted images were first linearly aligned to the ICBM 152 average template using a 9-parameter transformation (3 translations, rotations, and scales)^74^ and preprocessed to minimize the effects of intensity non-uniformity^75^. Images were then classified to gray matter (GM), white matter (WM) and cerebrospinal fluid^76^. The hemispheres were modeled as GM and WM surfaces using a deformable model strategy that generates four separate surfaces defined by 40,962 vertices each^77^. CT was derived between homologous vertices on GM and WM surfaces, and was derived using the t-link metric and subsequently blurred with a 28.28-mm surface-based diffusion kernel^78^. Native-space thicknesses were used in all analyses reported^79,80^. Homology across the population was achieved through non-linear surface-based normalization that uses a mid-surface (between pial and WM surfaces)^81^. This normalization uses a depth-potential function^82^ that fits each subject to a minimally biased surface-based template^83^. All vertex-wise analyses were performed in the RMINC package (https://wiki.phenogenomics.ca/display/MICePub/RMINC) and were corrected for multiple comparisons using the false discovery rate (FDR)^84^. All vertex-wise statistics were carried out using a general linear model (GLM) that included age and sex as covariates.

### Anatomical correlations analysis (MACACC)

**Figure 6:**
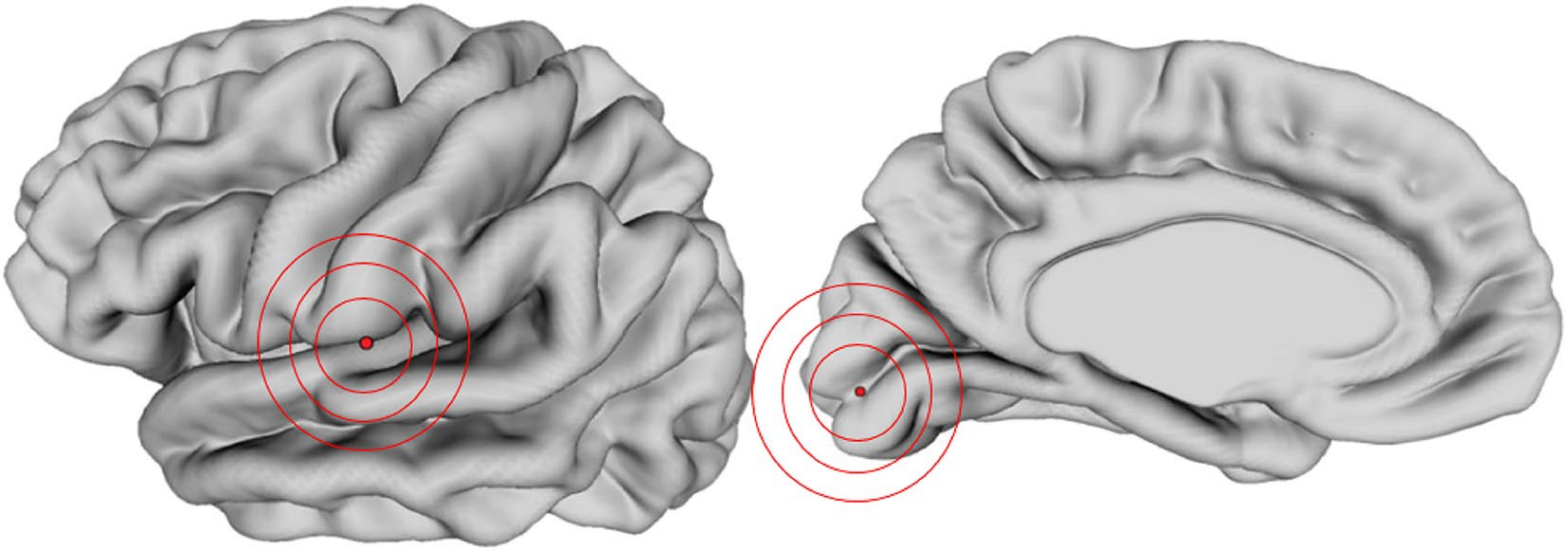
Seed locations are shown here in the left hemisphere, marked with a red dot. The corresponding vertex point was used in the right hemisphere.

In order to detect cortical thickness (CT) relationships between visual and auditory areas, we used the “Mapping anatomical correlations across cerebral cortex (MACACC) analysis”^26^, also known as seed-based structural covariance, which is performed by correlating the cortical thickness of a seed vertex of interest with the CT of all other brain vertices. For this analysis, we chose two seeds per hemisphere, representative of auditory and visual modalities: Heschl’s gyrus and V1 (for vertex point localization, see Table 1, Fig. 6, and Supplementary Information, Supplementary Materials and Methods). We then correlated the CT value of each seed vertex against the CT value of all other vertices in the brain, for each subject and group separately. To find if the MACACC maps were different between groups, we used the MACACC-slope statistic shown in Lerch and colleagues^26^. All maps were FDR-corrected at 10%.

**Table 1:** Seed locations.

Seed	x	y	z	Vertex point
(a) Heschl’s gyrus	52	− 12	2	11,296
(b) V1	10	− 90	4	33,028

Coordinates in MNI. Same vertex point for both hemispheres.

### DTI preprocessing, processing, and statistics

The DTI images were converted from DICOM to NIFTI format. Then, we corrected for susceptibility-induced off-resonance field, movement and eddy currents distortion using the DTI sequence (blip-down) and the DTIPA sequence (blip-up) in the FSL’s “eddy” toolbox^85^. Afterwards, we obtained the mean FA images from each subject created by fitting a tensor model to the raw diffusion data using FDT, and then brain-extracted using BET^86^. All subjects’ FA data were then aligned into standard space using the nonlinear registration tool FNIRT^87–89^. Voxel-wise statistical analysis of the FA data was carried out using *tract-based spatial statistics* (TBSS)^90,91^. Briefly, the mean FA image was created and thinned to create the mean FA skeleton that represents the centers of all tracts common to the group. The mean FA skeleton was further thresholded at an FA value of 0.2 to exclude peripheral tracts. Each subject’s aligned FA maps were then projected onto the mean FA skeleton and the resulting data were fed into voxel-wise statistics.

For the statistical analysis, we first created a ROI mask from the JHU-WM tractography atlas in MNI space^92–94^ and the mean FA skeleton mask only from the left and right inferior fronto-occipital fasciculus (IFOF). We decided on that particular tract because there is evidence that it connects auditory and visual areas^30^. The statistical analysis was performed by the FSL toolbox “randomize” using 5000 random permutations. We first contrasted both groups (NM vs. MUS). Then, to assess the relationship between behaviorally measured bimodal compatibility gain (BCG) and FA values across all participants, we correlated the BCG variable with mean FA in the ROI mask voxels. Age, gender, and group were included in the model as covariates. Threshold-free cluster enhancement (TFCE)^95^ was used to control for multiple comparisons by using family-wise error (FWE) at alpha = 0.05. From the resulting significant clusters, we extracted the individual mean FA for further analysis. Motivated by previous exploratory analyses of our behavioral data^25^, we repeated this process on the two groups separately. In this analysis, control for multiple comparisons was achieved by using family-wise error (FWE) at the more liberal alpha = 0.10 because of the reduced sample sizes and hence statistical power. The TBSS results were inflated using tbss_fill only for visualization purposes. Coordinates are shown in MNI space at peak cluster value.

## Supplementary Information

Supplementary Information.
