# Supplementary material for: Audiovisual structural connectivity in musicians and non-musicians: a cortical thickness and diffusion tensor imaging study
Source: Sci Rep. 2021 Feb 22;11:4324. doi: 10.1038/s41598-021-83135-x (PMC7900203; doi:10.1038/s41598-021-83135-x)
Supplement: Supplementary file 1 — Supplementary Information. [file 41598_2021_83135_MOESM1_ESM.pdf]

SUPPLEMENTARY INFORMATION TO:

Audiovisual structural connectivity in musicians and non-musicians: A cortical thickness and diffusion tensor imaging study

Cecilie Møller*+, Eduardo A Garza-Villarreal*+, Niels Chr. Hansen, Andreas Højlund, Klaus B Bærentsen, M. Mallar Chakravarty, Peter Vuust

+*these authors contributed equally to this work*

Supplementary Information

Supplementary Introduction

This MRI/DTI study is part of a larger study that also includes magnetoencephalography recorded in a separate session, a full behavioral experiment[1], a test of musical aptitude[2], a custom-made test of pitch direction sensitivity, and the Autism-spectrum Quotient (AQ)[3]. Participants took part in all assessments. In addition to the MRI/DTI data, only the measure of bimodal compatibility gain (BCG) derived from the behavioral experiment, the pitch discrimination threshold estimation, and data collected in the online questionnaire, Goldsmiths Musical Sophistication Index, v.1.0[4] are relevant to the current investigation, and hence described in the present manuscript.

Supplementary Materials and Methods: Behavioral experiment

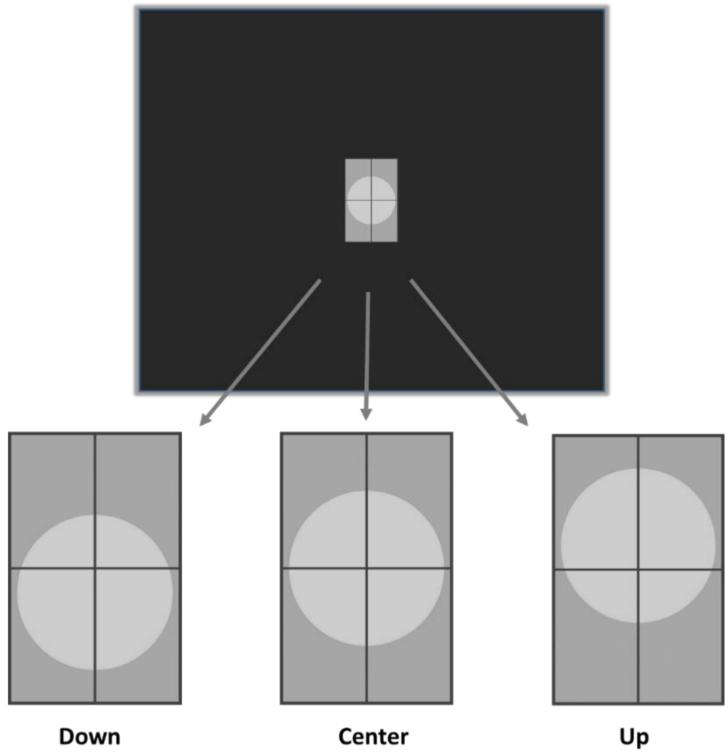

Figure S1. Visual stimuli. A pitch deviant could be presented simultaneously with either one of these three images. Crossmodally matching deviants were audiovisual stimulus pairs of which the subcomponents were going in the same direction relative to the standard (i.e., a high pitch deviant coupled with “up”/ a low pitch deviant coupled with “down”). Responses to these were compared against responses to audiovisual stimulus pairs in which no visual cue to the pitch change was presented, (i.e., a pitch change coupled with “Center”). This difference was denoted the *bimodal compatibility gain* (BCG).

Supplementary Materials and Methods: Anatomical Correlations Analysis (MACACC)

To find seeds with a functional significance, we derived them using the Neurosynth meta-analytic tool (http://neurosynth.org) searching for the functional maps related to the specific terms (Heschl's gyrus and V1). We downloaded the FDR-corrected maps, used FSL "cluster" tool with a threshold of Z = 1 to extract clusters, and we localized the peak value coordinate of the greatest significant cluster in MNI. That coordinate was used to find the equivalent vertex "point" in the surface maps using the toolbox "Display" (the same point for both hemispheres) (see Table 1, Fig. 6 of the main text).

Supplementary Results: MACACC

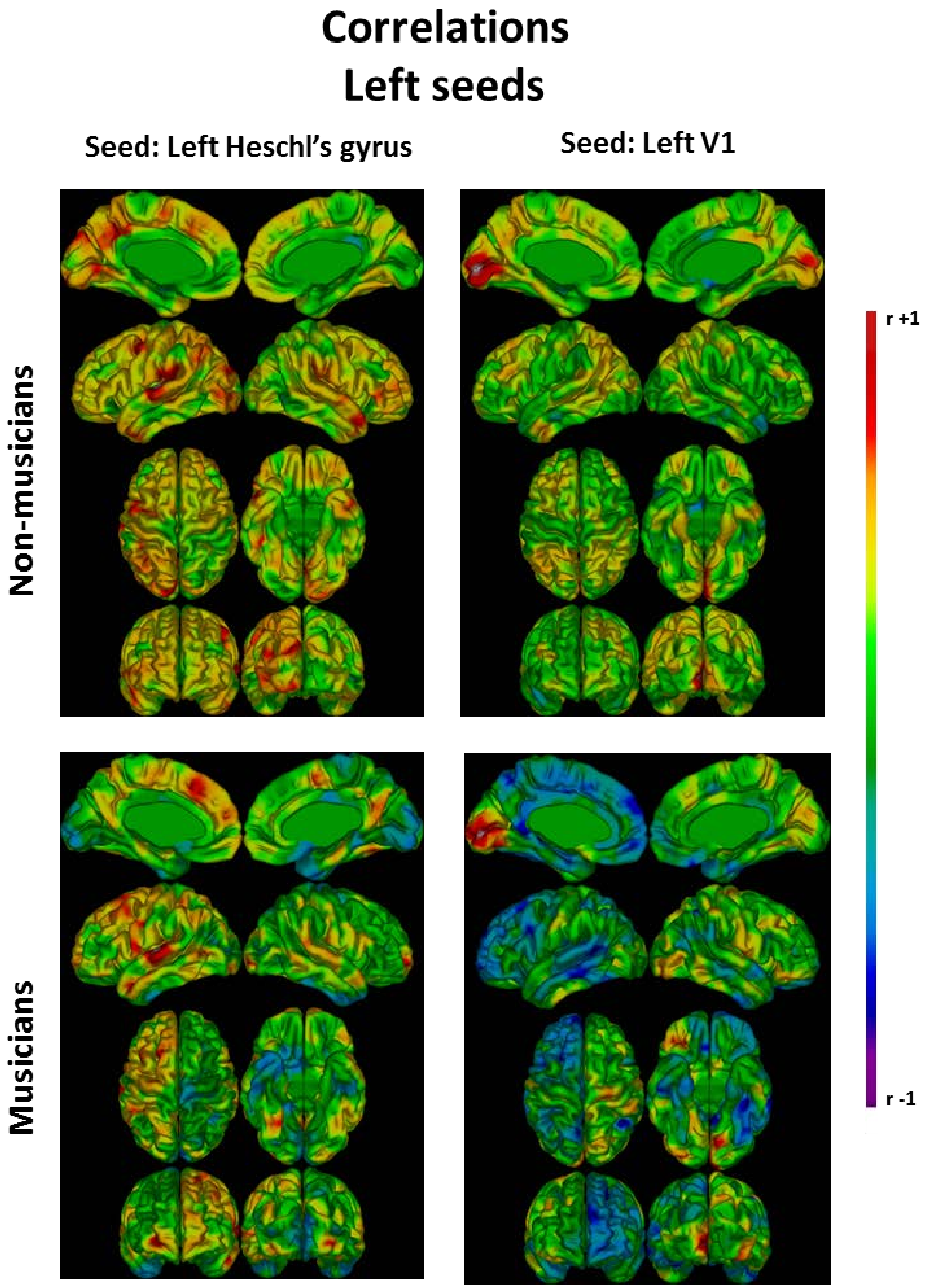

Figure S2. All correlations underlying the MACACC-results presented in Fig. 3 of the main text.

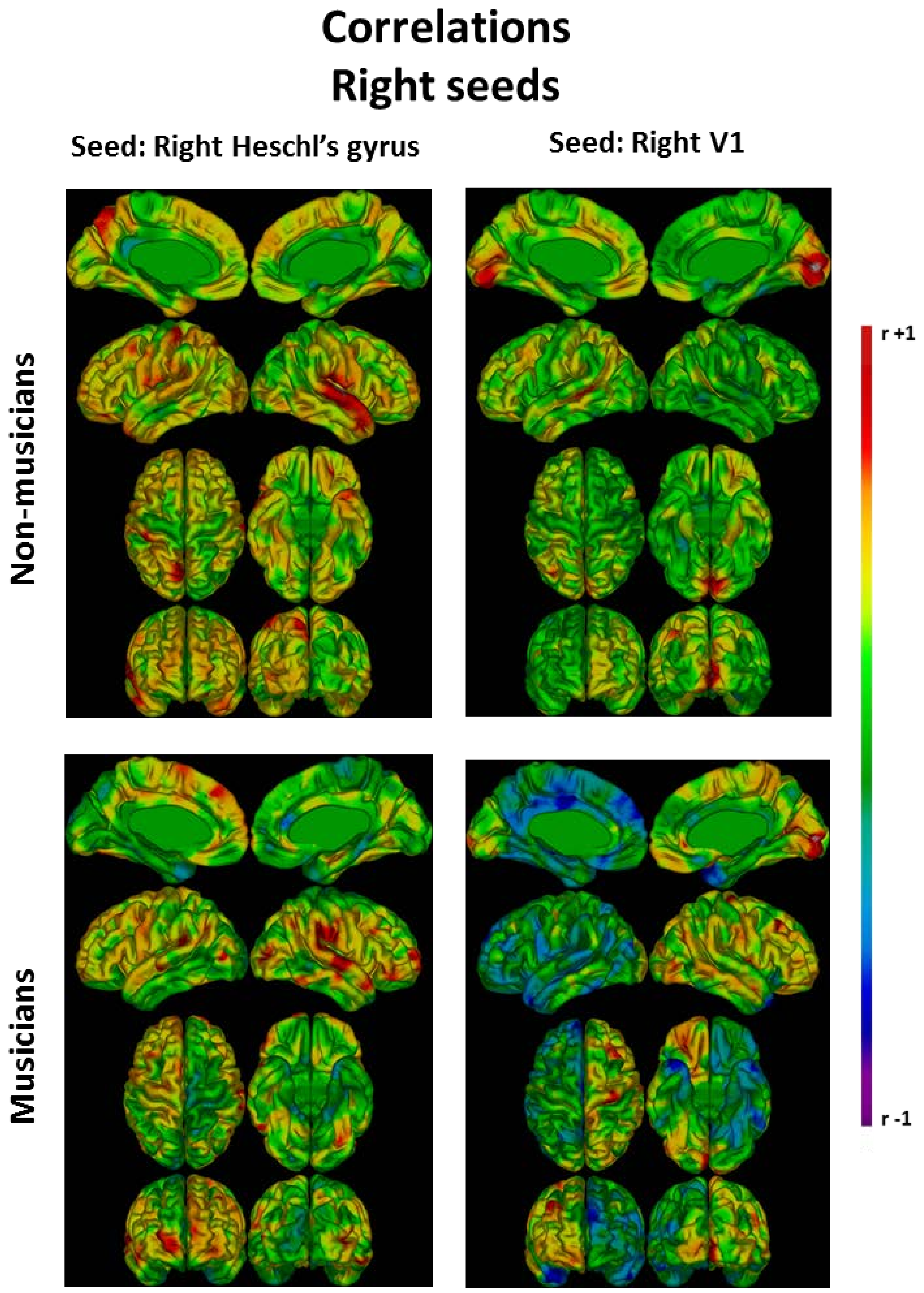

Figure S3. All correlations underlying the MACACC-results presented in Fig. 4 of the main text.

Supplementary Results: MACACC

Table S1. Non-local areas of significant cortical thickness correlation in non-musicians and musicians.

**NON-MUSICIANS**	*x*	*y*	*z*	*t-value*
**Heschl's seed left**				
Left Hemisphere				
Precentral gyrus	-48	2	45	5.29
Lingual gyrus	-1	-70	3	5.12
Supramarginal gyrus	-61	-35	26	5.93
Angular gyrus	-56	-59	36	4.69
Temporal Pole	-52	6	-40	4.66
Frontal Pole	-47	40	11	3.34
Frontal Pole	-27	65	13	3.14
Middle Temporal gyrus	-61	-33	-3	4.41
Occipital pole	-12	-96	29	5.74
Lateral occipital cortex, superior division	-37	-89	24	5.07
Occipital pole	-9	-100	-11	5.15
Lateral occipital cortex, inferior division	-41	-90	0	4.55
Precuneus	-8	-50	39	5.13
Juxtapositional Lobule Cortex	-3	-6	57	4.41
Paracyngulate gyrus	-3	21	43	4.25
Orbitofrontal cortex	-8	40	-25	3.70
Right Hemisphere				
Supramarginal gyrus, anterior division	60	-20	27	4.44
Middle Temporal gyrus, anterior division	60	1	2	4.37
Temporal pole	50	10	-27	5.96
Inferior Frontal gyrus, pars triangularis	56	30	-1	4.58
Frontal pole	42	54	9	4.17
Middle Frontal gyrus	40	24	49	3.33
Frontal pole	28	45	37	3.6
Inferior Temporal gyrus, posterior division	47	-36	-24	5.52
Occipital pole	24	-96	25	3.46
**Heschl's seed right**				
Right hemisphere				
Frontal Operculum Cortex	42	26	2	5.77
Frontal Pole	27	65	6	3.98
Temporal Fusiform Cortex, posterior division	36	-29	-30	4.00
Lateral Occipital Cortex	48	-70	-8	3.84
Postcentral Gyrus	37	-35	54	3.49
Occipital Fusiform Gyrus	26	-63	-8	4.47
Precuneous Cortex	1	-49	59	3.7
Juxtapositional Lobule Cortex (SMA)	6	-7	49	3.69
Precuneous Cortex	3	-43	54	3.64
Precuneous Cortex	1	-63	21	3.62
Superior Frontal Gyrus	5	49	32	3.46
Lateral Occipital Cortex, superior division	32	-63	51	3.39

Left hemisphere				
Postcentral Gyrus	-44	-29	66	5.46
Precentral Gyrus	-63	-1	17	4.88
Middle Frontal Gyrus	-42	14	49	4.87
Precentral Gyrus	-57	-1	44	4.42
Lateral Occipital Cortex, inferior division	-39	-90	3	4.47
Occipital Pole	-27	-95	9	3.73
Precuneous Cortex	-1	-72	46	5.74
Cuneal Cortex	-4	-74	26	4.04
Precentral Gyrus	-63	-3	17	4.92
Frontal Pole	-26	64	4	4.18
Frontal Pole	-22	42	-20	5.40
Temporal Pole	-41	15	-39	4.89
Occipital Fusiform Gyrus	-20	-76	-7	3.92
Superior Frontal Gyrus	-11	33	59	3.43
Frontal Medial Cortex	-3	35	-16	2.96
Paracingulate Gyrus	-3	26	44	3.05
Precuneous Cortex	-3	-43	46	4.19
Lingual Gyrus	-7	-60	-1	3.17
**V1 seed left**				
Left hemisphere				
Planum Temporale	-58	-38	19	3.23
Lateral Occipital Cortex, superior division	-45	-60	35	3.59
Lateral Occipital Cortex, superior division	-40	-70	51	3.40
Middle Temporal Gyrus, temporooccipital part	-53	-60	7	3.61
Superior Temporal Gyrus, posterior division	-54	-29	-2	3.01
Inferior Temporal Gyrus, anterior division	-52	-5	-32	3.88
Middle Frontal Gyrus	-45	30	24	3.43
Superior Frontal Gyrus	-21	30	52	3.52
Superior Parietal Lobule	-13	-53	74	3.20
Juxtapositional Lobule Cortex (SMA)	-3	-6	57	3.21
Precuneous Cortex	-5	-52	40	3.67
Precuneous Cortex	-17	-61	19	4.21
Parahippocampal Gyrus, posterior division	-21	-37	-17	3.21
Frontal Pole	-31	45	-17	3.43
Right hemisphere				
Occipital Pole	1	-90	11	5.27
**V1 seed right**				
Left hemisphere				
Lingual Gyrus	-5	-84	-13	6.51
Frontal Medial Cortex	-4	42	-13	3.83
Cingulate Gyrus, anterior division	-1	1	40	4.07
Precuneous Cortex	-8	-55	50	3.32
Frontal Pole	-26	40	-18	4.82
Middle Frontal Gyrus	-45	19	41	3.66
Supramarginal Gyrus, posterior division	-50	-44	6	6.03
Lateral Occipital Cortex, superior division	-37	-73	48	4.67
Angular Gyrus	-45	-58	36	3.73

Lateral Occipital Cortex, superior division	-17	-68	51	3.95
Parahippocampal Gyrus, posterior division	-33	-25	-21	3.21
**MUSICIANS**	*x*	*y*	*z*	*t-value*
**Heschl's seed left**				
**Heschl's seed right**				
**V1 seed left**				
**V1 seed right**				
Right Hemisphere				
Middle Frontal Gyrus	39	27	47	5.53

Notes. Significant t-values at FDR 10%. The coordinates of the brain areas are identical to the significant findings depicted in Fig. 3 and Fig. 4 of the main text, except that only non-local areas are included in the table.

Supplementary Discussion

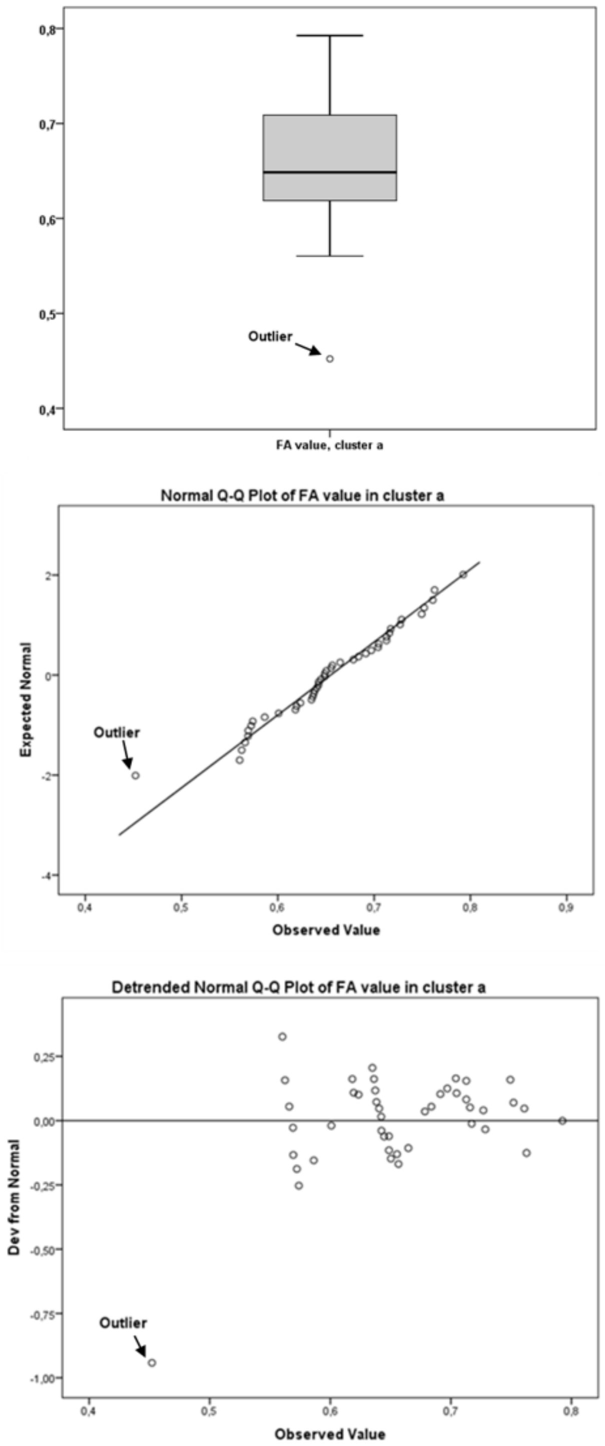

Figure S4. Outlier diagnostics. The FA of one participant (marked here as "Outlier") was 2.956 standard deviations lower than the mean FA of all participants.

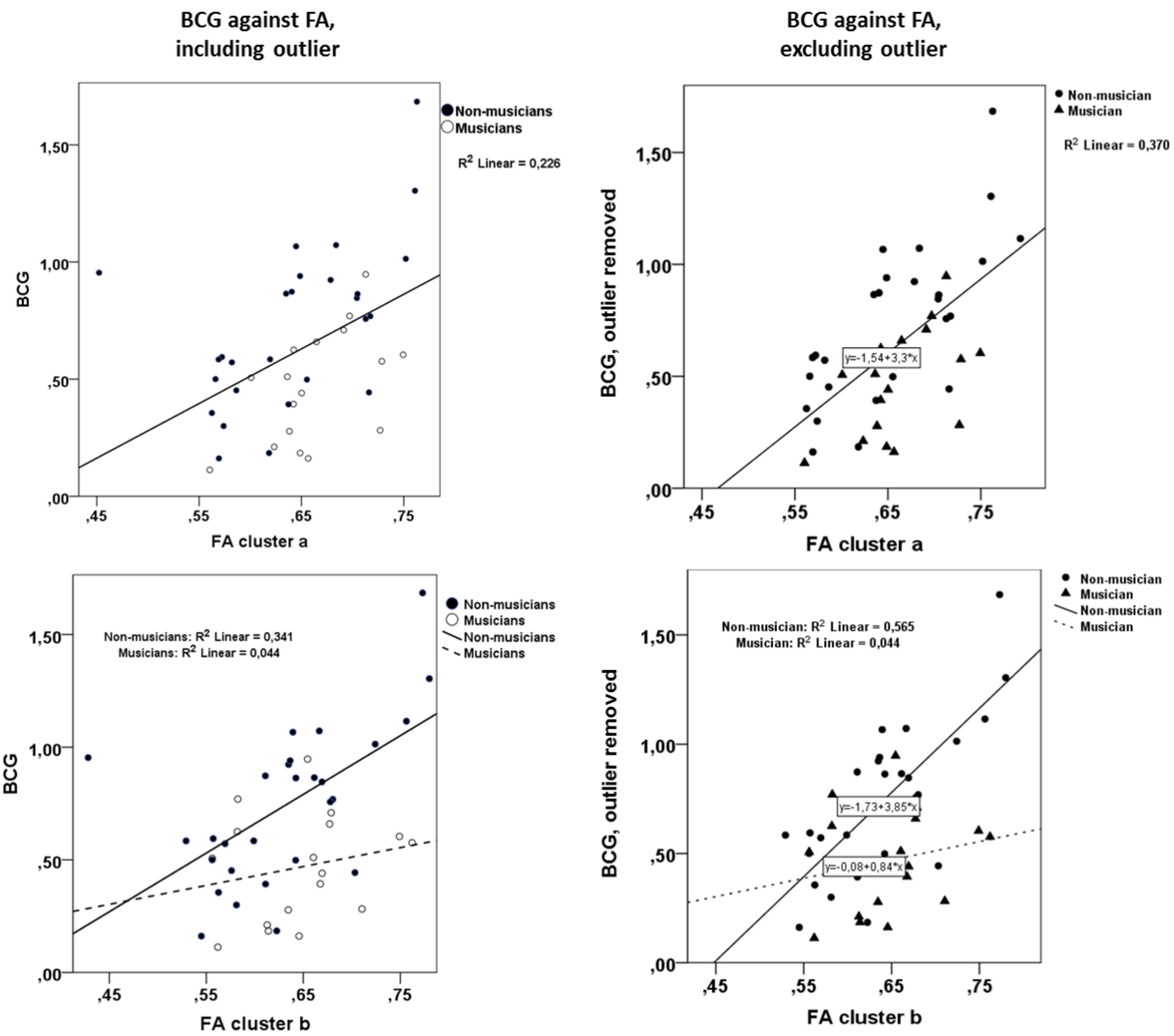

Figure S5. Bimodal compatibility gain plotted against mean FA of the voxels in the significant clusters. Plots on the right show the effect of removing the outlier identified in Fig. S4. It is unlikely that removing this outlier would have had a negative influence on the results reported in the main text.

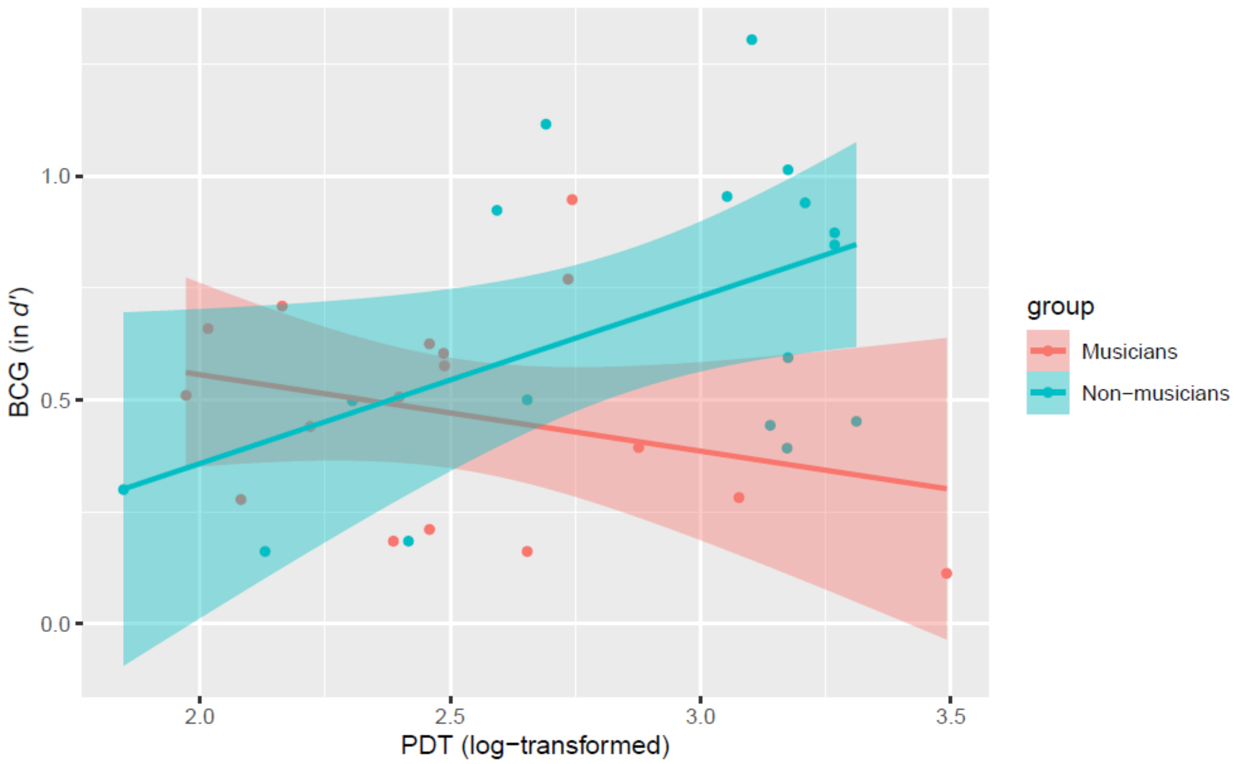

Figure S6. Pitch discrimination thresholds (PDT) plotted against bimodal compatibility gain (BCG) in musicians (n = 17) and an n-matched subgroup of non-musicians (n = 17) consisting of participants whose PDTs were within the same range as the PDTs of musicians.

Supplementary References

1. Møller, C. *et al.* Visually induced gains in pitch discrimination: Linking audio-visual processing with auditory abilities. *Attention, Perception, & Psychophysics* **80**, 999–1010 (2018).
2. Wallentin, M., Nielsen, A. H., Friis-Olivarius, M., Vuust, C. & Vuust, P. The Musical Ear Test, a new reliable test for measuring musical competence. *Learning and Individual Differences* **20**, 188 196 (2010).
3. Baron-Cohen, S., Wheelwright, S., Skinner, R., Martin, J. & Clubley, E. The autism-spectrum quotient (AQ): evidence from Asperger syndrome/high-functioning autism, males and females, scientists and mathematicians. *J.Autism Dev.Disord.* **31**, 5 (2001).
4. Müllensiefen, D., Gingras, B., Musil, J. & Stewart, L. The Musicality of Non-Musicians: An Index for Assessing Musical Sophistication in the General Population. *PLoS ONE* **9**, e89642 (2014).
